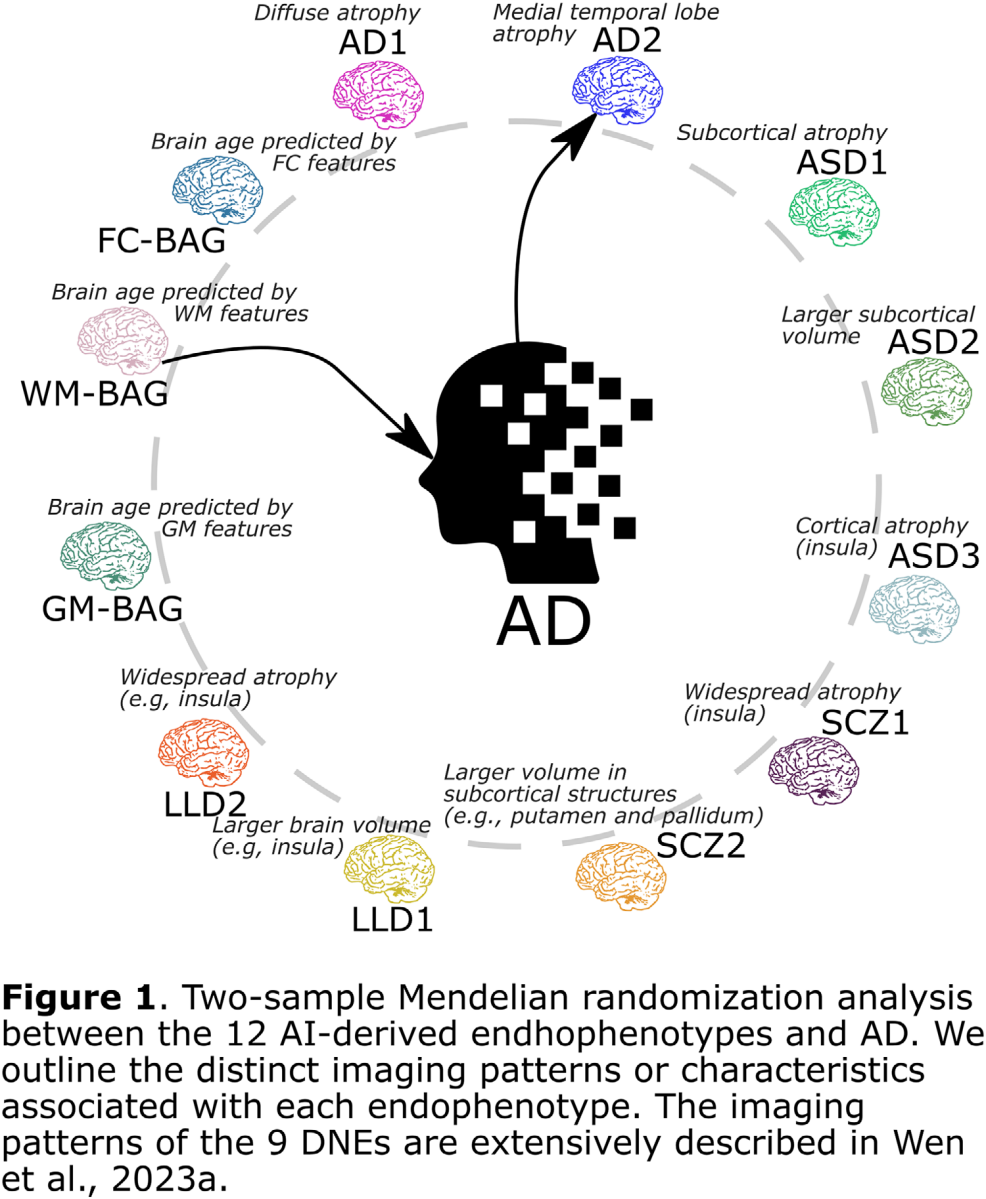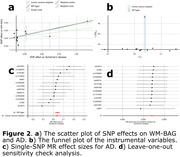# AI‐derived endophenotypes are causally associated with Alzheimer's disease

**DOI:** 10.1002/alz.093673

**Published:** 2025-01-09

**Authors:** Junhao Wen, Arthur W. Toga, Christos Davatzikos

**Affiliations:** ^1^ University of Southern California, LA, CA USA; ^2^ Laboratory of Neuro Imaging, Stevens Neuroimaging and Informatics Institute, Keck School of Medicine, University of Southern California, Los Angeles, CA USA; ^3^ Artificial Intelligence in Biomedical Imaging Laboratory (AIBIL), Perelman School of Medicine, University of Pennsylvania, Philadelphia, PA USA

## Abstract

**Background:**

Brain morphology changes due to both natural aging and various pathological conditions. We used magnetic resonance imaging (MRI) and artificial intelligence (AI) to derive three brain age gaps (Wen et al., 2023b) [gray matter (GM), white matter (WM), and functional connectivity (FC)‐BAG] for brain aging and 9 dimensional neuroimaging endophenotypes (Wen et al., 2023a) [DNE: Alzheimer's disease (AD)1‐2, autism spectrum disorder (ASD)1‐3, schizophrenia (SCZ)1‐2, and late‐life depression (LLD)1‐2) for disease heterogeneity (a.k.a., subtypes). We applied genome‐wide association study (GWAS) summary statistics of the 3 BAGs, 9 DNEs, and two AD case‐control studies (Lambert et al., 2013; Wightman et al., 2021) to decipher the potential causality between these AI‐derived endophenotypes and AD (Fig. 1).

**Method:**

We investigated whether the 3 BAGs and 9 DNEs (AD1‐2, ASD1‐3) were causally linked to AD using a bi‐directional two‐sample Mendelian randomization implemented in the TwoSampleMR R package (Hemani et al., 2018) with five different estimators. No individual data were used. The GWAS summary statistics of the 3 BAGs and 9 DNEs were performed using the UK Biobank data publicly available at https://labs.loni.usc.edu/medicine. The AD summary statistics are obtained at https://pgc.unc.edu/for‐researchers/download‐results/ for Wightman et al. and at https://gwas.mrcieu.ac.uk/ for Lambert et al., excluding the UK Biobank participants.

**Result:**

We identified potential causal relationships from AD2, characterized by medial temporal lobe atrophy, to AD [P‐value=1.74x10‐4, OR (95% CI) = 1.25 (1.11, 1.40), number of SNPs=7], and AD to WM‐BAG [P‐value=7.18x10‐5, OR (95% CI) = 1.04 (1.02, 1.05), number of SNPs=13] (Fig. 1). Regarding the latter, our sensitivity check indicated the absence of apparent outlier SNPs or horizontal pleiotropy (Verbanck et al., 2018) (Egger estimator intercept: ‐2.22x10‐3±3.87810‐3, P‐value=0.57). All five estimators demonstrated consistent causal effects. The funnel plot displayed no noticeable asymmetry. Most IVs exhibited positive effects on AD, with three showing a negative association. Additionally, leave‐one‐out analyses indicated that no single IV dominated this causal relationship (Fig. 2).

**Conclusion:**

This investigation revealed a causal association between two AI‐derived endophenotypes and AD. These endophenotypes could be used to identify individuals at high risk of developing AD and select populations in future AD clinical trials.